# Prediction of residual stones after ESWL for ureteral calculi using deep learning-radiomics-based stone heterogeneity and body composition: an interaction analysis

**DOI:** 10.3389/fmed.2026.1932507

**Published:** 2026-10-14

**Authors:** Shumin Wei, Qingqing Fang, Lin Zhang, Liwei Zhou

**Affiliations:** 1 Medical Imaging Department, Xinghua People’s Hospital Affiliated to Yangzhou University, Xinghua City, Jiangsu, China; 2 Department of Urology, Xinghua People’s Hospital Affiliated to Yangzhou University, Xinghua City, Jiangsu, China

**Keywords:** body composition, deep learning, extracorporeal shock wave lithotripsy, interaction analysis, residual stone

## Abstract

**Objective:**

To investigate the association between deep learning-radiomics-based stone heterogeneity, body composition, and residual stones after extracorporeal shock wave lithotripsy (ESWL) for ureteral calculi.

**Methods:**

This retrospective study included 200 patients with ureteral stones who underwent ESWL between January 2024 and December 2025, comprising the development cohort. An additional 57 patients treated between January 2026 and June 2026 were included as the temporal testing cohort. Baseline clinical data and imaging data were collected. Deep learning (DL) and radiomics features of ureteral stones were extracted, and feature selection was performed to construct a DL-radiomics model (Radscore). TotalSegmentator was used to quantify visceral fat, skeletal muscle, and subcutaneous fat volumes within the T12-S4 region. The predictive performance of the clinical-body composition model, DL-radiomics model, and nomogram model was compared. Subgroup analyses were conducted to evaluate the stability of the nomogram model. The interaction between the Radscore and body composition in relation to residual stones was further explored using the optimal cutoff values derived from the training set.

**Results:**

The development cohort was randomly divided into a training set and a validation set in a 7:3 ratio. Visceral fat and skeletal muscle volumes were included in the clinical-body composition model, yielding AUCs of 0.792 (0.706, 0.878), 0.821 (0.577, 1.000) and 0.827 (0.710, 0.943), respectively. The DL radiomics model achieved AUCs of 0.778 (0.679−0.876), 0.704 (0.363−1.000) and 0.742 (0.547−0.937). The nomogram model showed AUCs of 0.893 (0.835, 0.952), 0.824 (0.630, 1.000) and 0.898 (0.807, 0.989). Subgroup analyses of stone location, side of the stone, and perinephritis revealed no significant interaction in the nomogram model (*P* > 0.05). Calibration and decision curve analyses demonstrated good agreement and clinical utility of the nomogram model. In the interaction analysis between Radscore and body composition, visceral fat volume significantly attenuated the risk of residual stones associated with a high stone Radscore.

**Conclusion:**

Deep learning-radiomics-based stone heterogeneity demonstrates good predictive performance for residual stones after ESWL. Moreover, lower visceral fat volume significantly attenuates the impact of stone heterogeneity on residual stone formation.

## 1 Introduction

Ureteral stones represent one of the most common types of urolithiasis, with an estimated prevalence of nearly 20% (1). Extracorporeal shock wave lithotripsy (ESWL) is widely used in clinical practice for the management of ureteral stones (2), owing to its noninvasive nature, ease of operation, good patient tolerance, and rapid postoperative recovery. According to the European Association of Urology guidelines, stones are categorized by size into three groups (<10 mm, 10−20 mm, and >20 mm), and ESWL is recommended as a primary treatment option for stones smaller than 20 mm (3). However, the reported success rates of ESWL vary considerably, ranging from 35% to 89% (4, 5). Therefore, identifying patients who are most likely to benefit from ESWL remains a key clinical challenge. Previous studies have shown that the efficacy of ESWL is influenced by multiple factors, including stone composition and structural characteristics, patient sex, skin-to-stone distance (SSD), and body mass index (6, 7). These findings suggest that treatment outcomes are determined not only by the intrinsic heterogeneity of the stone but also by the heterogeneity of the tissues along the energy transmission pathway.

Regarding intrastone heterogeneity, Mustafa et al. (8) demonstrated through texture analysis that parameters such as kurtosis, entropy, dissimilarity, and energy are important predictors of ESWL outcomes. With the development of radiomics and deep learning techniques, it has become increasingly feasible to characterize stone microstructure at a more detailed level (9, 10). In terms of body habitus and tissue composition, the SSD has been widely recognized as a factor influencing ESWL efficacy (11–13) However, this transmission pathway should not be considered a simple geometric distance. It consists of multiple tissue layers, including visceral fat (e.g., perirenal fat), skeletal muscle, subcutaneous fat, and skin. Treating this pathway as homogeneous may overlook the effects of acoustic impedance mismatches between different tissues, which can lead to energy scattering and attenuation. As shock waves propagate through these heterogeneous media, substantial energy loss may occur, thereby reducing treatment effectiveness. Furthermore, Juan et al. (14) identified visceral fat and perirenal fat as independent risk factors for ESWL failure, supporting the role of surrounding tissue attenuation in treatment outcomes. Although previous studies have acknowledged the influence of fat and muscle components, their assessment often relies on manual measurements, which are time-consuming and subject to inter-observer variability, limiting their practical application in routine clinical settings.

In light of the above, the present study aimed to systematically evaluate the predictive roles of stone heterogeneity and body composition in residual stone formation following ESWL for ureteral calculi. The specific objectives were as follows: (1) to quantify stone heterogeneity using a combined deep learning and radiomics approach and assess its association with ESWL outcomes; (2) to automatically segment abdominal visceral fat, skeletal muscle, and subcutaneous fat using TotalSegmentator (15), and examine their relationships with post-ESWL residual stones; (3) to develop a predictive model integrating features of stone heterogeneity and body composition, and to further investigate their potential interaction effects on treatment outcomes. The study was conducted in accordance with the Imaging Biomarker Standardization Initiative (IBSI) and the TRIPOD + AI reporting guidelines (see Supplementary Materials S1 and S2).

## 2 Methods

### 2.1 Patients and outcome definition

This retrospective study included patients with ureteral stones who underwent ESWL at our institution between January 2024 and December 2025 as the development cohort (*n* = 200). This development cohort was randomly divided into a training set (*n* = 140) and a validation set (*n* = 60) in a 7:3 ratio. An independent temporal testing cohort consisted of 57 consecutive patients treated between January 2026 and June 2026. The inclusion criteria were as follows: (1) first-time diagnosis of ureteral stones confirmed by computed tomography (CT) or intravenous urography, with a stone diameter ≤ 1 cm; (2) no history of ureteral stone surgery or ESWL. The exclusion criteria were as follows: (1) multiple ureteral stones on the same side; (2) prior open ureteral surgery or a history of ureteral stricture; (3) absence of post-treatment imaging follow-up; (4) incomplete clinical data.

All ESWL procedures were performed using an HK.ESWL-V extracorporeal shock wave lithotripter (Wikkon, China) following a strictly standardized institutional protocol. To minimize treatment-related confounding factors, the operational parameters were maintained uniformly across all patients: an energy setting of 13.0−14.0 kV, a shock wave frequency of 60 shocks per minute (1 Hz), and a total of 1,500 to 2,000 shock waves per session. For analgesia, a standardized preoperative regimen of non-steroidal anti-inflammatory drugs was administered. The clinical endpoint in this study was evaluated strictly based on the outcome of the initial (first) ESWL session. All patients underwent abdominal CT follow-up within two weeks after this single session. Residual stone was defined as the presence of fragments larger than 3 mm on this specific follow-up imaging (14). The study complied with the Declaration of Helsinki and was approved by our institutional ethics committee (XRYLL-KY-2026002).

### 2.2 Clinical variables and CT acquisition

The following clinical variables were collected: age, gender, history of hypertension, diabetes, and cardiovascular disease, perinephritis, hydronephrosis, maximum stone diameter, mean stone density, SSD, side of the stone, stone location, ureteral wall thickness, proximal and distal ureteral diameters.

All patients underwent CT scanning using a 64-slice CT scanner (LightSpeed VCT, GE Healthcare). The scan range extended from 2 cm above the diaphragm to the superior border of the pubic symphysis. Scanning parameters were as follows: tube voltage, 120 kV; automatic tube current modulation; pitch, 0.8; collimation, 128 × 0.625 mm. Based on the raw data, thin-slice images were reconstructed with a slice thickness of 1.25 mm and an interval of 1.25 mm.

### 2.3 Deep learning and radiomics feature extraction

Deep learning features were extracted using a U-Net-based architecture integrated with a channel transformer module (16, 17) (Figure 1). Each downsampling and upsampling block consisted of two grouped convolutional layers, each comprising a 3 × 3 convolution, followed by batch normalization and a ReLU activation function. The network was trained exclusively on the training set (*n* = 140) using a composite loss function combining binary cross-entropy (0.5) and Dice loss (0.5). Model training was performed using the Adam optimizer with an initial learning rate of 0.0001, a batch size of 32, L2 regularization, and 50 training epochs. During the training process, the validation set (*n* = 60) was used to monitor performance, and the model weights achieving the lowest loss on the validation set were retained for feature extraction. The temporal testing cohort (*n* = 57) was completely locked out of this phase and strictly reserved for final independent evaluation.

**Figure 1:**
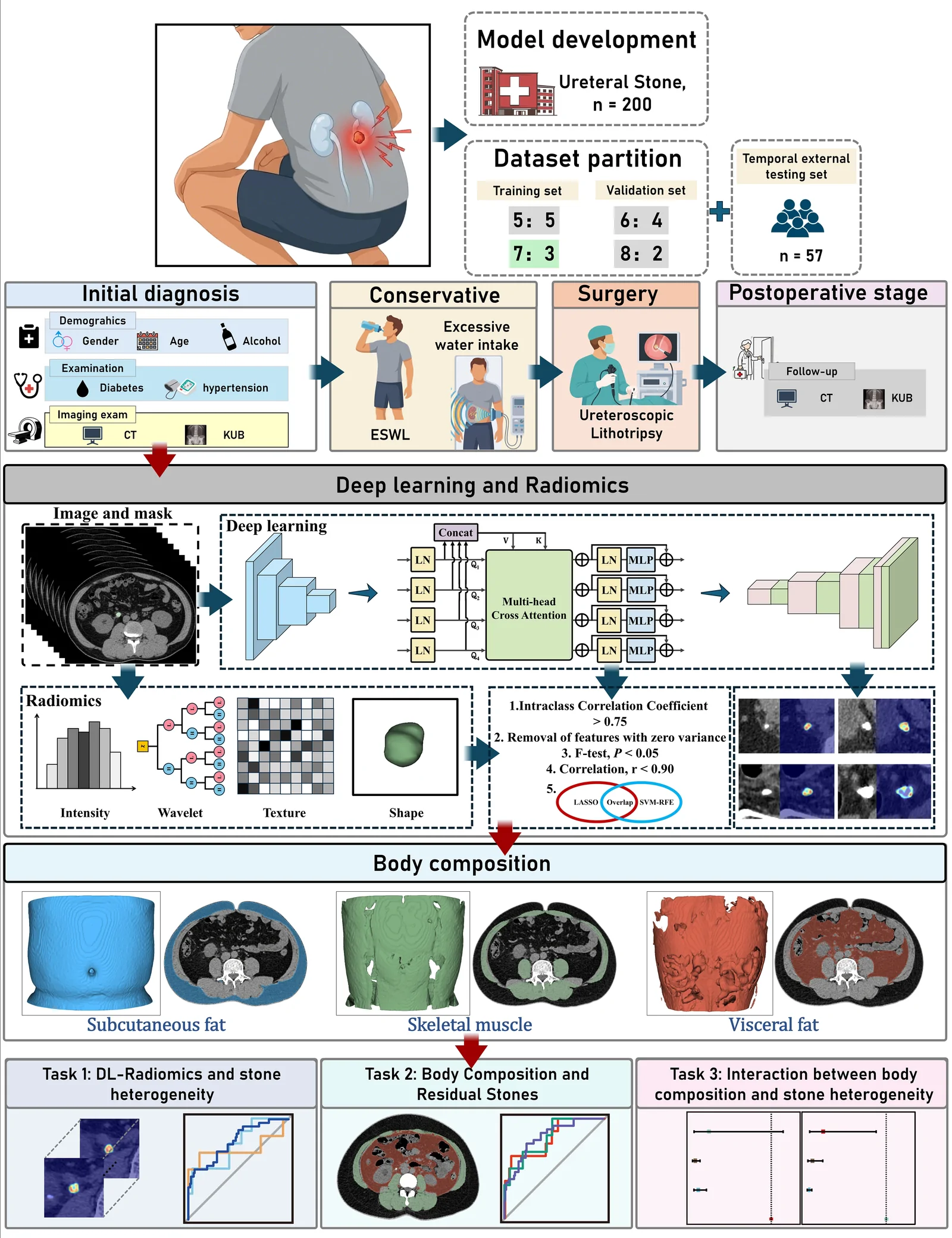
Overview of the workflow for radiomics and DL-radiomics feature extraction, model development, feature selection, body composition quantification, and result analysis.

The workflow for deep learning feature extraction was as follows: (1) A rectangular region of interest (ROI) encompassing the stone was manually defined and resized to 224 × 224 pixels as the network input; (2) Image intensities were normalized using min-max scaling; (3) The normalized images were fed into the trained network, and feature maps were extracted from the fourth downsampling layer of the U-Net encoder; (4) Global average pooling was applied to obtain a 1 × 512-dimensional feature vector for each image; (5) An unsupervised clustering approach was used to group features, followed by similarity-based selection to identify the most informative feature subsets. In total, 512 deep learning features were extracted for each patient and used for subsequent analyses. The model was implemented using PyTorch (version 2.3.1) with CUDA 11.8 and executed on an NVIDIA RTX 2080 Ti GPU.

ROIs of the stones were manually delineated using 3D Slicer (version 5.10.0). Images were resampled to isotropic voxels (1 × 1 × 1 mm^3^) using B-spline interpolation with a bin width of 25. ROI masks were resampled using nearest-neighbor interpolation. Global gray-level normalization was disabled to preserve the original physical meaning and absolute zero point of the image intensities. Radiomics features were extracted using the PyRadiomics package, including shape features, first-order statistics, gray-level co-occurrence matrix (GLCM), and gray-level run length matrix (GLRLM) features. To enhance feature representation, multiple image filters and transformations were applied, including original images, wavelet decomposition, Laplacian of Gaussian (LoG) filtering. In total, 851 radiomics features were extracted for subsequent analysis. ROI delineation was performed by a radiologist with 5 years of experience. To assess segmentation consistency, 60 patients were randomly selected from the training set and independently re-segmented by a second radiologist with 15 years of experience. These data were used for subsequent feature selection and stability assessment. Interobserver spatial overlap was quantified using the Dice similarity coefficient (DSC).

### 2.4 Statistical analysis

Statistical analyses were performed using R Studio (version 4.2.3) and Python (version 3.9.13). Continuous variables were expressed as mean ± standard (x*±s*) deviation and compared using one-way analysis of variance (ANOVA). Categorical variables were presented as counts (n) and percentages (%), and compared using the chi-square test. All multiple comparisons were adjusted using the Bonferroni correction. Dataset partitioning was conducted using the createDataPartition function in R.

The DL-radiomics feature selection process was conducted in isolation to prevent data leakage. Scaling parameters (mean and standard deviation) were calculated exclusively from the training set (*n* = 140) and subsequently applied unchanged to the validation and temporal test sets. All feature selection procedures, statistical tests, and coefficient estimations were performed exclusively using the training set (*n* = 140) (Supplementary Figure S1 and Supplementary Material S3). The detailed steps were as follows: (1) DL-radiomics Features extracted from ROIs independently delineated by two radiologists were evaluated using the intraclass correlation coefficient (ICC) based on the 60 patients sampled from the training set. Only features with ICC > 0.75 were retained; (2) In the training set (*n* = 140), DL-radiomics features with an ICC > 0.75 were Z-score standardized using scaling parameters, and zero-variance features were excluded; (3) DL-radiomics Features showing statistical significance in the *F*-test (*P* < 0.05) within the training set were retained; (4) Highly correlated redundant DL-radiomics features were removed using a correlation threshold of |r| > 0.9; (5) The intersection of the DL-radiomics feature subsets selected by LASSO regression (using 10-fold nested cross-validation within the training set) and SVM-RFE was used for final feature selection and dimensionality reduction. The 10-fold nested cross-validation provided an unbiased estimate of the internal AUC (Supplementary Figure S2). Finally, the intersected DL-radiomics features were integrated using a logistic regression model fitted only on the training set. The resulting weighting coefficients were locked to construct the Radscore formula (Figure 1). The finalized Radscore formula and cutoffs were directly applied to the validation and temporal testing cohorts.

Clinical variables and body composition parameters were first screened using the LASSO regression, followed by multivariable logistic regression to identify independent predictors of RS after ESWL. The predictive performance of the clinical model, body composition model, Radscore model, and combined model was subsequently compared, and confusion matrix analysis was performed to evaluate classification performance. A nomogram was constructed to facilitate individualized risk prediction. Subgroup analyses were conducted to assess the stability and consistency of the nomogram model. In addition, optimal cut-off values for body composition parameters and the Radscore were determined using the Youden index or clinically established criteria, and interactions between the Radscore and body composition features were further evaluated. The AUCs and 95% CIs in the validation and test sets were calculated using 1,000 bootstrap resampling iterations. Net reclassification improvement (NRI) and integrated discrimination improvement (IDI) analyses were subsequently performed to evaluate the incremental diagnostic value provided by the incorporation of the Nomogram model. *P* value < 0.05 was considered statistically significant.

## 3 Results

### 3.1 Baseline characteristic

A total of 257 patients were included in this study, with an age range of 18-92 years and a mean age of 48.54 ± 15.06 years. Residual stones were observed in 42 patients. Significant differences were observed between the two groups in age, sex, hypertension status, maximum stone diameter, mean stone density, distal ureteral diameter, ureteral wall thickness, skeletal muscle volume, visceral fat volume, and Radscore (all *P* < 0.05; Supplementary Table S1). Detailed baseline characteristics of the three groups are presented in Table 1.

**Table 1:** Baseline characteristics of the training, validation and testing sets.

Variables	Training set (*n* = 140)	Validation set (*n* = 60)	Testing set (*n* = 57)	*P*
Residual Stone (%)	28 (20.00)	6 (10.00)	8 (14.04)	0.185
Age (years)	48.72 ± 15.17	49.17 ± 14.43	47.46 ± 15.66	0.812
Gender (%)				0.531
Female	29 (20.71)	9 (15.00)	13 (22.81)	
Male	111 (79.29)	51 (85.00)	44 (77.19)	
Hypertension (%)				0.341
No	134 (95.71)	60 (100.00)	55 (96.49)	
Yes	6 (4.29)	0 (0.00)	2 (3.51)	
Diabetes (%)				0.281
No	135 (96.43)	55 (91.67)	53 (92.98)	
Yes	5 (3.57)	5 (8.33)	4 (7.02)	
Cardiovascular Disease (%)				0.813
No	137 (97.86)	59 (98.33)	57 (100.00)	
Yes	3 (2.14)	1 (1.67)	0 (0.00)	
Perinephritis (%)				0.611
No	74 (52.86)	35 (58.33)	34 (59.65)	
Yes	66 (47.14)	25 (41.67)	23 (40.35)	
Hydronephrosis (%)				0.126
No	14 (10.00)	5 (8.33)	1 (1.75)	
Yes	126 (90.00)	55 (91.67)	56 (98.25)	
Maximum stone diameter (mm)	4.92 ± 2.21	5.37 ± 3.87	4.63 ± 2.15	0.317
Mean stone density (HU)	664.94 ± 363.47	621.70 ± 310.85	647.19 ± 310.51	0.710
Skin-to-stone distance (mm)	107.33 ± 15.16	107.07 ± 14.83	106.81 ± 15.04	0.975
Side of the stone (%)				0.570
Left	76 (54.29)	28 (46.67)	28 (49.12)	
Right	64 (45.71)	32 (53.33)	29 (50.88)	
Stone location (%)				0.876
Lower ureter	52 (37.14)	21 (35.00)	24 (42.11)	
Mid ureter	19 (13.57)	8 (13.33)	9 (15.79)	
Upper ureter	69 (49.29)	31 (51.67)	24 (42.11)	
Proximal ureter diameter (mm)	7.04 ± 2.67	6.90 ± 2.65	7.00 ± 3.30	0.950
Distal ureteral diameter (mm)	5.55 ± 2.05	6.13 ± 2.81	5.64 ± 1.89	0.231
Ureteral wall thickness (mm)	3.82 ± 1.53	3.88 ± 1.54	3.90 ± 1.45	0.876
Skeletal muscle (cm^3^)	3,271.77 ± 893.31	3,261.67 ± 796.31	3,212.81 ± 761.03	0.904
Visceral fat (cm^3^)	3,336.81 ± 1,444.65	3,256.87 ± 1,209.96	3,456.57 ± 1,400.97	0.734
Subcutaneous fat (cm^3^)	4,083.37 ± 1,942.30	3,675.43 ± 1,985.02	4,208.04 ± 1,747.19	0.267
Radscore	−1.51 ± 1.73	−1.38 ± 2.40	−1.76 ± 1.36	0.516

### 3.2 Feature selection and radscore construction

After ICC-based filtering, 710 DL-radiomics features were retained, including 479 handcrafted radiomics features and 231 deep learning features. Subsequent feature selection reduced the number of features to 612 after removal of zero-variance features (403 radiomics and 209 deep learning features), 568 after *F*-test filtering (381 radiomics and 187 deep learning features), and 313 after correlation filtering (154 radiomics and 159 deep learning features). After feature selection using both LASSO and SVM-RFE, the overlapping feature subset was retained, comprising four PyRadiomics features and one deep learning feature. The Radscore was calculated as follows:

$$Radscore=\;\beta_{0}\;+\;\beta_{1}F_{1}+\;\beta_{2}F_{2}+\;\beta_{3}F_{3}+\;\beta_{4}F_{4}+\;\beta_{5}F_{5}$$

In the final model, β_0_ represented the intercept (−1.5064). F1 corresponded to original_shape_Maximum2DdiameterRow with a coefficient (β_1_) of −0.0186. F2 represented wavelet-HLH_firstorder_InterquartileRange with a coefficient (β_2_) of 0.2107. F3 corresponded to DL_160 (DL imaging feature index No. 160) with a coefficient (β_3_) of 0.2824. F4 represented wavelet-HHL_glszm_SizeZoneNonUniformity with a coefficient (β_4_) of 1.5144. F5 represented wavelet-LLL_glcm_ClusterProminence with a coefficient (β_5_) of 0.4419. The DSC was 0.912 (interquartile range: 0.870-0.937). The unbiased internal mean AUC expectedly adjusted to 0.671 (Supplementary Figure S2).

### 3.3 Feature selection and performance evaluation for all models

In the training cohort, 28 patients with residual stones were included. According to the events-per-variable (EPV) principle, 3-5 features could be incorporated (18). LASSO regression was performed in the training set for variable selection. The model demonstrated optimal performance when eight predictors were included (Supplementary Figure S3), namely sex, hypertension, maximum stone diameter, mean stone density, distal ureteral diameter, ureteral wall thickness, skeletal muscle volume, and visceral fat volume. Subsequent multivariable logistic regression identified ureteral wall thickness, skeletal muscle volume, and visceral fat volume as independent predictors of residual stones after ESWL (Table 2). These variables were therefore used to construct the combined clinical-body composition model.

**Table 2:** Multivariable logistic regression analysis of clinical and body composition parameters.

Variable	B	S.E	*P*	OR	OR 95%*CI*	
					Lower	Upper
Gender						
Female	Ref					
Male	−0.530	0.758	0.485	0.589	0.131	2.665
Hypertension						
No	Ref					
Yes	1.490	1,065	0.162	4.439	0.580	44.045
Maximum stone diameter	0.177	0.168	0.290	1.194	0.861	1.669
Mean stone density	0.001	0.001	0.137	1.001	0.999	1.003
Distal ureteral diameter	0.270	0.144	0.062	1.309	1.012	1.804
Ureteral wall thickness	0.424	0.188	0.003	1.527	1.074	2.261
Skeletal muscle	−0.001	0.001	0.014	0.999	0.998	0.999
Visceral fat	0.001	0.001	0.003	1.001	1.000	1.001

As shown in Table 3 and Figure 2, the clinical-body composition model achieved an AUC of 0.792 (95% CI: 0.706, 0.878) in the training set, with a sensitivity of 0.929 and specificity of 0.571. In the validation set, the AUC was 0.821 (95% CI: 0.577, 1.000), with a sensitivity of 0.667 and specificity of 0.981. In the testing set, the model yielded an AUC of 0.827 (95% CI: 0.710, 0.943), with a sensitivity of 1.000 and specificity of 0.673. The DL radiomics model showed an AUC of 0.778 (95% CI: 0.679, 0.876) in the training set, with a sensitivity of 0.500 and specificity of 0.938. In the validation set, the AUC was 0.704 (95% CI: 0.363, 1.000), with a sensitivity of 0.667 and specificity of 0.889, while in the testing set, the AUC reached 0.742 (95% CI: 0.547, 0.937), with a sensitivity of 0.625 and specificity of 0.898. The combined model (nomogram) demonstrated the best overall performance. It achieved an AUC of 0.893 (95% CI: 0.835, 0.952) in the training set, with a sensitivity of 0.893 and specificity of 0.759. In the validation set, the AUC was 0.824 (95% CI: 0.630, 1.000), with a sensitivity of 0.667 and specificity of 0.889. In the testing set, the model yielded an AUC of 0.898 (95% CI: 0.807, 0.989), with a sensitivity of 1.000 and specificity of 0.694. The nomogram model showed relatively better performance, although not significantly better than the individual models, in terms of ROC curves, calibration performance, and decision curve analysis (Figure 3 and Supplementary Table S2). Continuous NRI and IDI analyses demonstrated a statistically significant incremental benefit of the nomogram model over the clinical–body composition model in the training cohort, whereas no significant incremental benefit was observed in the validation or test cohorts (Supplementary Table S3).

**Table 3:** Predictive performance of different models.

Dataset	AUC (95%CI)	Accuracy	Sensitivity (95%CI)	Specificity (95%CI)	PPV (95%CI)	NPV (95%CI)	*P*_Delong_
Clinical-body composition model							
Training	0.792 (0.706, 0.878)	0.643	0.929 (0.821, 1.000)	0.571 (0.473, 0.661)	0.351 (0.301, 0.414)	0.970 (0.925, 1.000)	-
Validation	0.821 (0.577, 1.000)	0.950	0.667 (0.333, 1.000)	0.981 (0.944, 1.000)	0.800 (0.500, 1.000)	0.964 (0.929, 1.000)	-
Testing	0.827 (0.710, 0.943)	0.719	1.000 (1.000, 1.000)	0.673 (0.551, 0.816)	0.333 (0.258, 0.444)	1.000 (1.000, 1.000)	-
DL-Radiomics model							
Training	0.778 (0.679, 0.876)	0.850	0.500 (0.306, 0.694)	0.938 (0.875, 0.975)	0.667 (0.430, 0.854)	0.882 (0.810, 0.934)	0.839
Validation	0.704 (0.363, 1.000)	0.867	0.667 (0.223, 0.957)	0.889 (0.774, 0.958)	0.400 (0.122, 0.738)	0.960 (0.863, 0.995)	0.585
Testing	0.742 (0.547, 0.937)	0.860	0.625 (0.245, 0.915)	0.898 (0.778, 0.966)	0.500 (0.187, 0.813)	0.936 (0.825, 0.987)	0.510
Nomogram model							
Training	0.893 (0.835, 0.952)	0.786	0.893 (0.750, 1.000)	0.759 (0.679, 0.830)	0.481 (0.404, 0.574)	0.966 (0.929, 1.000)	0.011
Validation	0.824 (0.630, 1.000)	0.867	0.667 (0.333, 1.000)	0.889 (0.796, 0.963)	0.400 (0.200, 0.714)	0.960 (0.918, 1.000)	0.956
Testing	0.898 (0.807, 0.989)	0.737	1.000 (1.000, 1.000)	0.694 (0.571, 0.816)	0.348 (0.276, 0.471)	1.000 (1.000, 1.000)	0.170

AUC, area under the receiver operating characteristic curve; PPV, positive predictive value; NPV, negative predictive value. DeLong's test was used to compare the DL-Radiomics model and Nomogram model with the Clinical–body composition model in each dataset (training, validation, and test sets).

**Figure 2:**
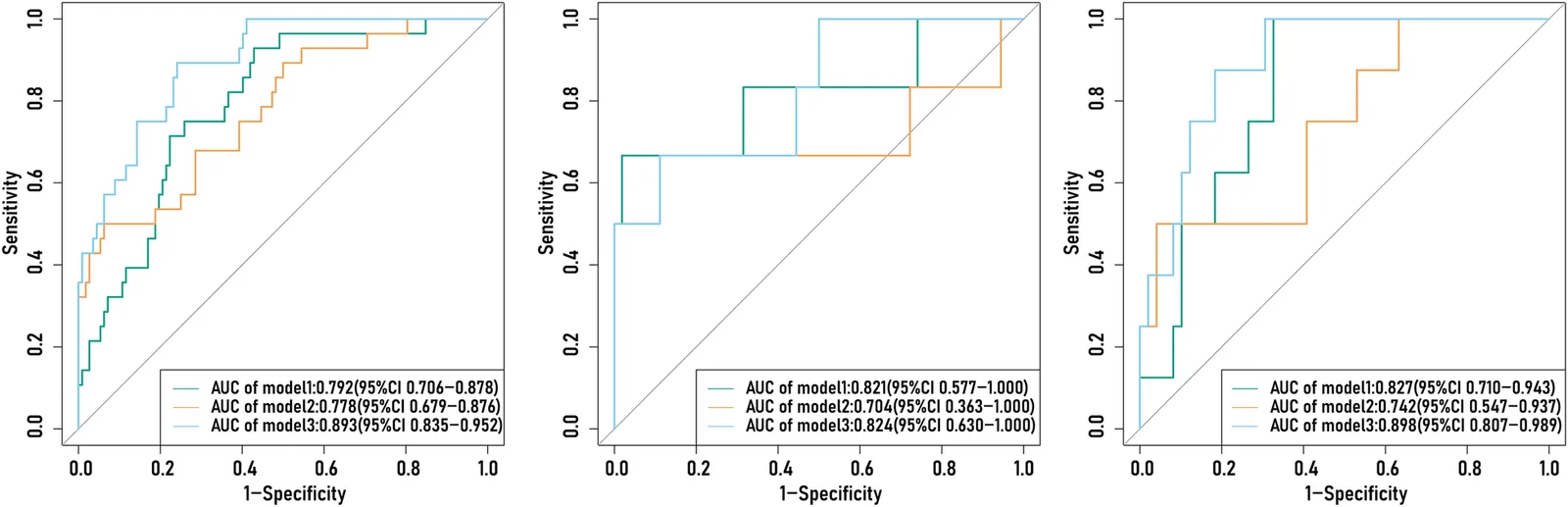
Receiver operating characteristic (ROC) curves of the clinical-body composition model, DL-radiomics model, and nomogram model in the training, validation, and testing sets. Model 1: Clinical-Body Composition model; Model 2: DL-Radiomics model; Model 3: Nomogram model.

**Figure 3:**
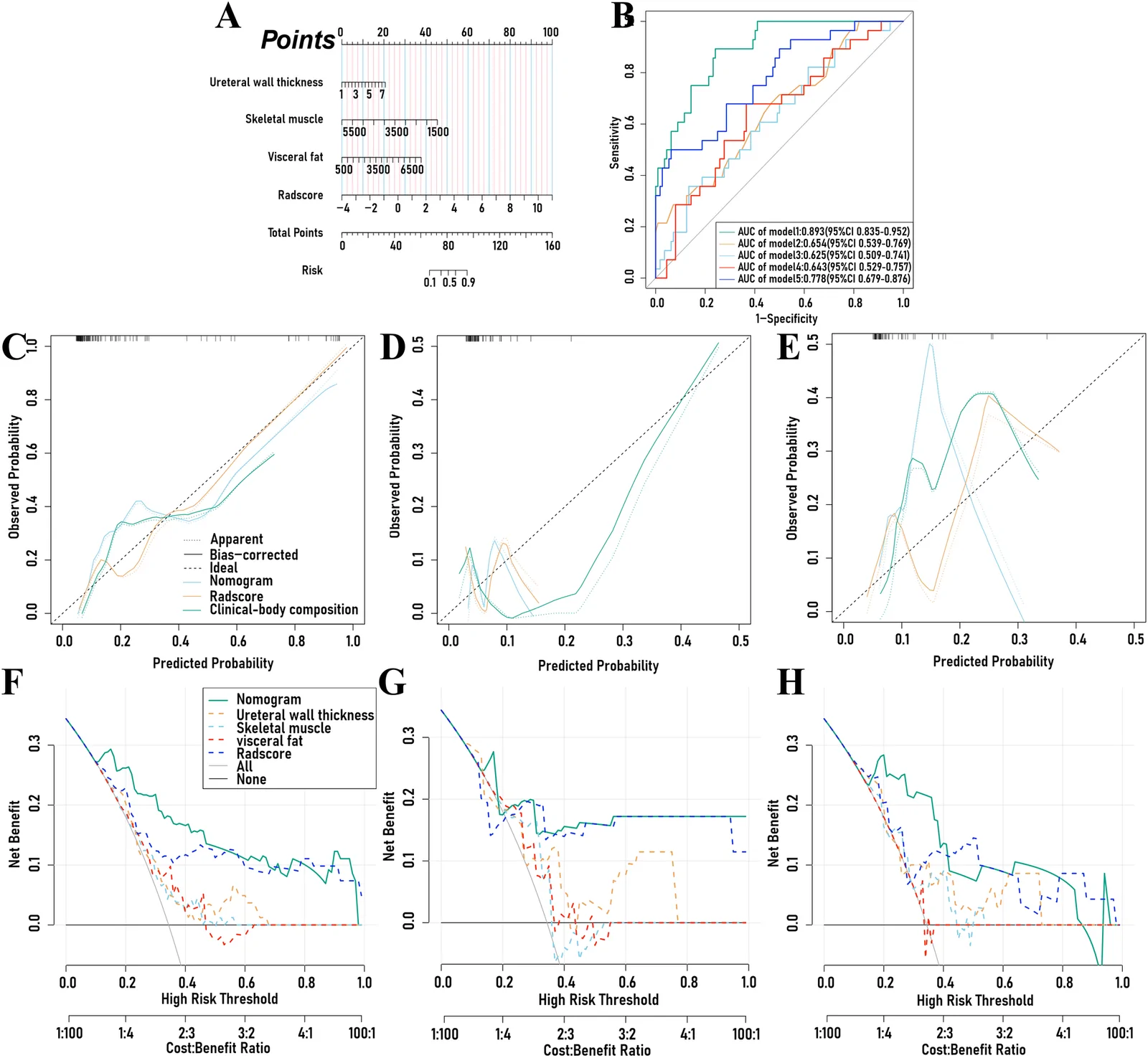
Nomogram model, single-feature ROC curves, calibration curves, and decision curve analysis (DCA). **(A)** Nomogram model. **(B)** ROC curves of the nomogram model and individual features in the training set: Model 1, nomogram model; Model 2, ureteral wall thickness; Model 3, skeletal muscle; Model 4, visceral fat; Model 5, DL-radiomics model. **(C–E)** ROC curves for the training, validation, and testing sets. **(F–H)** DCA curves for the training, validation, and testing sets.

Subgroup analysis (Table 4) showed no significant interaction in model performance across stone location, perinephritis status, or stone laterality in either the training or test set (all *P* > 0.05).

**Table 4:** Subgroup analysis of the nomogram model.

Dataset	AUC (95%CI)	Accuracy	Sensitivity (95%CI)	Specificity (95%CI)	PPV (95%CI)	NPV (95%CI)	*P*_Interaction_
Training							
Stone location							0.313
Lower ureter	0.924 (0.836, 1.000)	0.885	0.714 (0.290, 0.963)	0.911 (0.788, 0.975)	0.556 (0.212, 0.863)	0.953 (0.842, 0.994)	
Mid ureter	0.923 (0.801, 1.000)	0.842	1.000 (0.541, 1.000)	0.769 (0.462, 0.950)	0.667 (0.299, 0.925)	1.000 (0.692, 1.000)	
Upper ureter	0.852 (0.744, 0.960)	0.696	0.933 (0.681, 0.998)	0.630 (0.487, 0.757)	0.412 (0.246, 0.593)	0.971 (0.851, 0.999)	
Perinephritis							0.851
No	0.887 (0.796, 0.977)	0.757	0.857 (0.572, 0.982)	0.733 (0.603, 0.839)	0.429 (0.245, 0.628)	0.957 (0.852, 0.995)	
Yes	0.898 (0.821, 0.976)	0.818	0.929 (0.661, 0.998)	0.788 (0.653, 0.889)	0.542 (0.328, 0.744)	0.976 (0.874, 0.999)	
Side of the stone							0.148
Left	0.846 (0.753, 0.939)	0.750	0.824 (0.566, 0.962)	0.729 (0.597, 0.836)	0.467 (0.283, 0.657)	0.935 (0.821, 0.986)	
Right	0.957 (0.909, 1.000)	0.828	1.000 (0.715, 1.000)	0.792 (0.659, 0.892)	0.500 (0.282, 0.718)	1.000 (0.916, 1.000)	
Testing							
Stone location							0.163
Lower ureter	0.952 (0.846, 1.000)	0.750	1.000 (0.292, 1.000)	0.714 (0.478, 0.887)	0.333 (0.075, 0.701)	1.000 (0.782, 1.000)	
Mid ureter	0.929 (0.731, 1.000)	0.889	1.000 (0.158, 1.000)	0.857 (0.421, 0.996)	0.667 (0.094, 0.992)	1.000 (0.541, 1.000)	
Upper ureter	0.825 (0.591, 1.000)	0.667	1.000 (0.292, 1.000)	0.619 (0.384, 0.819)	0.273 (0.060, 0.610)	1.000 (0.753, 1.000)	
Perinephritis							0.859
No	0.938 (0.802, 1.000)	0.676	1.000 (0.158, 1.000)	0.656 (0.468, 0.814)	0.154 (0.019, 0.454)	1.000 (0.839, 1.000)	
Yes	0.922 (0.811, 1.000)	0.826	1.000 (0.541, 1.000)	0.765 (0.501, 0.932)	0.600 (0.262, 0.878)	1.000 (0.753, 1.000)	
Side of the stone							0.706
Left	0.871 (0.721, 1.000)	0.714	1.000 (0.541, 1.000)	0.636 (0.407, 0.828)	0.429 (0.177, 0.711)	1.000 (0.768, 1.000)	
Right	0.907 (0.796, 1.000)	0.759	1.000 (0.158, 1.000)	0.741 (0.537, 0.889)	0.222 (0.028, 0.600)	1.000 (0.832, 1.000)	

AUC, area under the receiver operating characteristic curve; PPV, positive predictive value; NPV, negative predictive value.

### 3.4 Interaction between body composition features and radscore

Based on the Youden index, Radscore (−0.917), skeletal muscle volume (3560.578), and visceral fat volume (3645.864) were dichotomized into high and low groups. Interaction analyses showed that, compared with skeletal muscle volume, low visceral fat volume significantly attenuated the effect of a high Radscore on the risk of residual stones after ESWL. The lowest risk was observed in patients with both low visceral fat and low Radscore (Figures 4A, 5). In the interaction analysis between skeletal muscle volume and Radscore, skeletal muscle exhibited a relatively weaker protective effect on residual stone formation compared with the Radscore itself (Figures 4B, 5).

**Figure 4:**
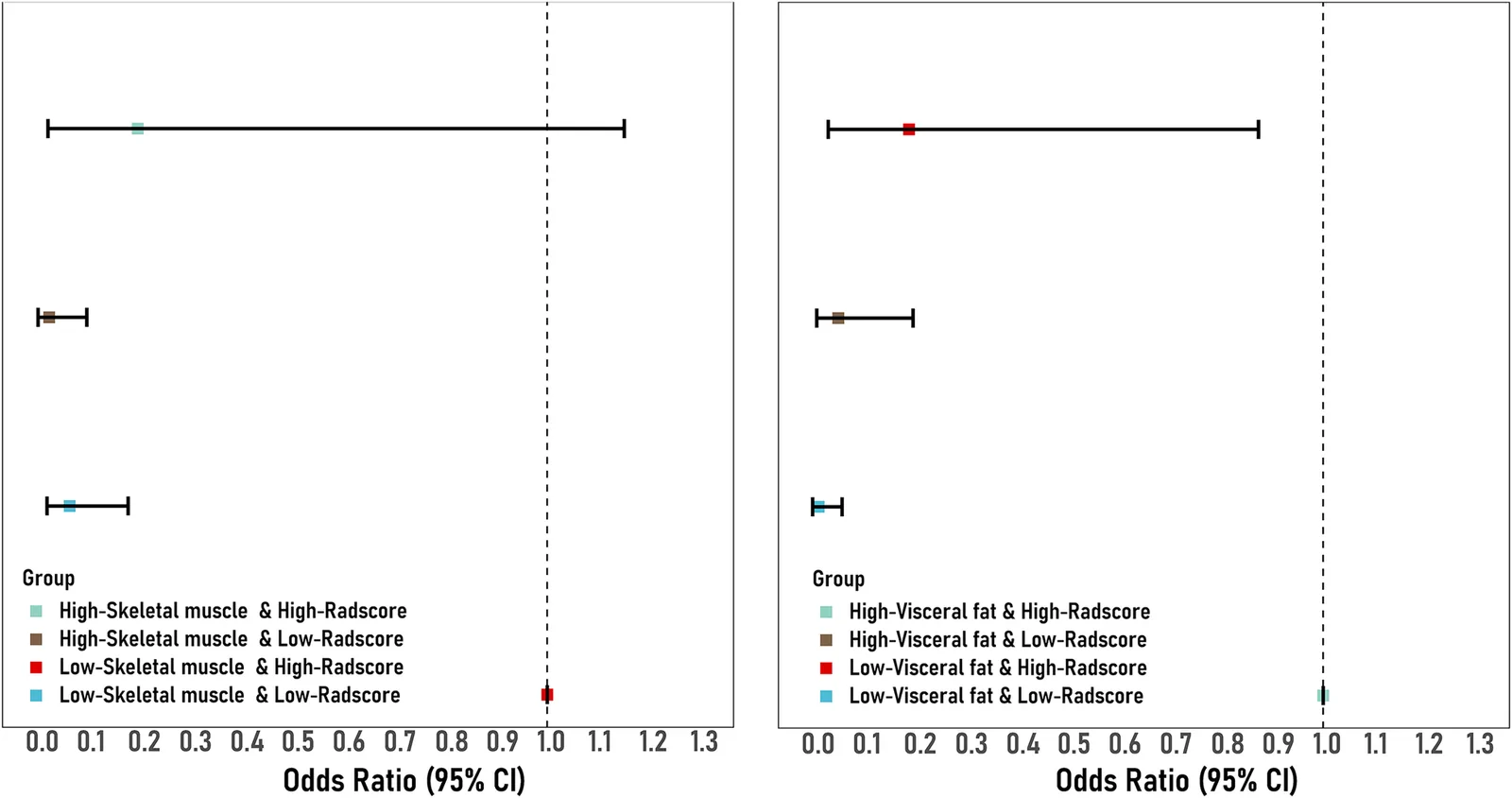
Forest plot illustrating the interaction effects of skeletal muscle and visceral fat with the radscore.

**Figure 5:**
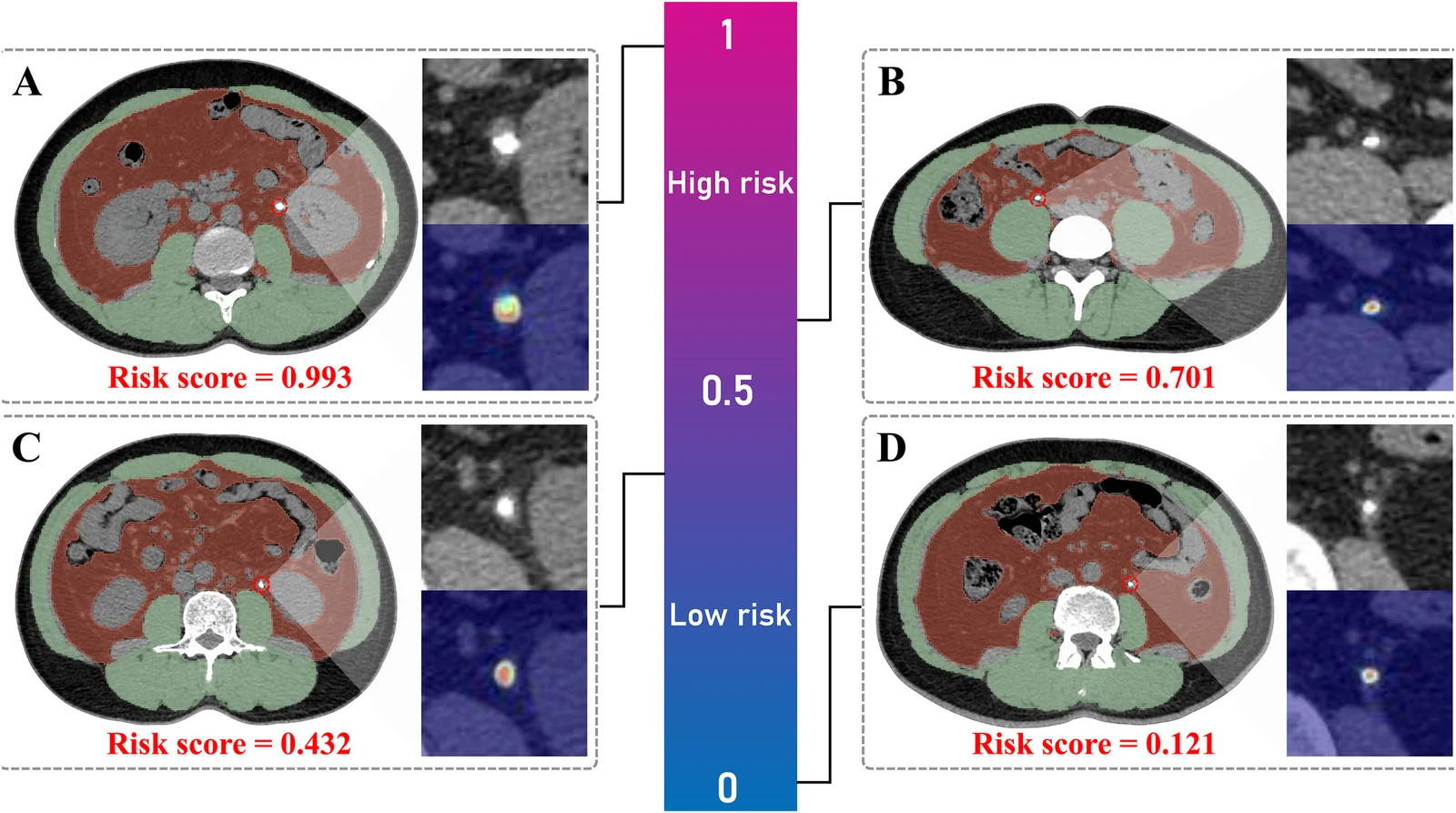
Grad-CAM visualization, skeletal muscle, and visceral fat across different risk score levels. (**A,B**) Visualization of the risk score, skeletal muscle, visceral fat, and Grad-CAM in patients with residual stones. (**C,D**) Visualization of the risk score, skeletal muscle, visceral fat, and Grad-CAM in patients with stone-free status.

## 4 Discussion

In this study, we evaluated the predictive value of ureteral stone characteristics and body composition features using a deep learning-radiomics approach for residual stone formation after ESWL. The DL radiomics model demonstrated good predictive performance (AUC = 0.704-0.778, 95%CI: 0.363-1.000), suggesting substantial intrinsic heterogeneity within stone properties at a microstructural level. Previous studies have reported that ureteral wall thickness, rather than stone size, location, or HU value, is the only independent risk factor for ESWL failure (13). In contrast, our findings indicate that body composition-related parameters, particularly visceral fat and skeletal muscle volume, may serve as stronger independent predictors than conventional anatomical distance-based measures. Based on these findings, we further investigated the interaction between Radscore and body composition features. Low visceral fat volume significantly attenuated the adverse effect of a high Radscore on the risk of residual stones after ESWL. In contrast, the protective effect of skeletal muscle volume was less consistent and was only observed in the low Radscore subgroup. However, these interaction findings must be interpreted with strict caution and viewed primarily as hypothesis-generating observations. To further explore the combined predictive value of imaging-derived stone characteristics and body composition, we constructed a nomogram model, which demonstrated robust performance in the training, validation and testing cohorts. Subgroup analyses showed no significant interaction across stone location, side of the stone, or perinephritis status.

The Radscore developed in this study integrates both macroscopic geometric features and microscopic textural heterogeneity of ureteral stones. Notably, SizeZoneNonUniformity demonstrated the highest predictive weight, suggesting that non-uniform spatial distribution within the stone matrix may be a key determinant of impaired energy transmission and incomplete fragmentation during ESWL. Compared with conventional metrics such as mean CT attenuation, which primarily reflect overall stone density, higher-order radiomics features provide more detailed information on the spatial complexity of internal crystal organization, thereby enabling a more refined characterization of stone structure. Previous studies have also explored stone heterogeneity. Arikan et al. (19) quantified the variation coefficient and standard deviation of stone density on non-contrast CT and reported that a higher variation coefficient was an independent protective factor for ESWL success (OR=0.998, 95% CI: 0.997-0.999). Similarly, Yamashita et al. (20) demonstrated that mean stone density derived from three-dimensional imaging reconstruction was also an independent predictor of ESWL outcomes. In contrast to previous radiomics studies, the present work identified SizeZoneNonUniformity from wavelet-transformed features as the most influential predictor. By comparison, Xu et al. (9) and Song et al. (21) reported wavelet-HLL_firstorder_Skewness as the dominant feature, whereas Yang et al. (22) identified wavelet-HLL_glcm_LMC2 as the most predictive variable. These discrepancies in selected features are likely attributable to differences in feature selection strategies across studies. Most prior work relied primarily on LASSO regression for dimensionality reduction, which, although effective in compressing feature space, may be limited in capturing complex nonlinear relationships among features. In contrast, the combined use of LASSO and SVM-RFE in the present study allows for more robust identification of highly discriminative, potentially nonlinear feature interactions. Furthermore, the inclusion of the DL_160 feature extracted via a convolutional neural network helps compensate for the limitations of handcrafted radiomics in representing complex imaging patterns. While the formal statistical tests (DeLong, NRI) in the temporal testing cohort did not yield a statistically significant incremental improvement when adding the Radscore to the clinical-body composition model, this is likely a consequence of low statistical power driven by the small event count (*n* = 8). Nevertheless, the numerical improvement in the AUC point estimate underscores the theoretical value of this multimodal approach. From a pathophysiological perspective, the clinical variables (ureteral wall thickness) capture the macroscopic inflammatory environment and anatomical impaction, while the Radscore decodes the microscopic crystalline heterogeneity that impedes shockwave energy transmission. Together, they provide a biologically plausible, mechanistically complementary framework that warrants further validation in larger, adequately powered external cohorts.

Çoban et al. (23) achieved excellent performance (AUC = 0.949) in predicting urinary stones using conventional clinical predictors, whereas our study explored, from a multimodal imaging phenotype perspective, the potential roles of stone heterogeneity and body composition. Pareek et al. (24) reported in 2005 that BMI and HU value are independent predictors of stone-free rates after ESWL. Subsequently, Juan et al. (14) further demonstrated that visceral and perirenal fat are significant independent risk factors for ESWL failure. However, ureteral wall thickness is not a pure anatomical parameter; rather, it likely reflects a composite effect of multiple surrounding tissues, including visceral fat, skeletal muscle, and subcutaneous fat. To further delineate these effects, we applied the TotalSegmentator tool to perform abdominal body composition analysis, quantifying visceral fat volume, skeletal muscle volume, and subcutaneous fat volume. Our results showed that, in addition to visceral fat, skeletal muscle volume was also an independent predictor of residual stones after ESWL, albeit with a protective effect. This discrepancy may be explained by the distinct acoustic and anatomical properties of different tissues. Visceral fat is irregularly distributed and often interposed between abdominal organs, forming multiple heterogeneous acoustic interfaces. As shock waves propagate through alternating low- and high-density media, nonlinear scattering and refraction may occur, leading to energy dissipation and reduced focusing efficiency at the stone surface, thereby increasing the likelihood of treatment failure. In contrast, skeletal muscle exhibits a more organized and relatively homogeneous structure. Due to its higher water content, muscle tissue has lower acoustic impedance (25), which allows shock waves to pass through with less scattering loss. In addition, anatomically, skeletal muscle is typically located along more peripheral or structurally stable transmission pathways, whereas visceral fat is more closely adjacent to the ureter, making its influence on shock wave propagation more direct. Although the volume of subcutaneous fat was quantified in our workflow, it was not retained in the final multivariable model. This finding further supports our hypothesis. Anatomically, subcutaneous fat is located superficially beneath the skin and mainly serves as a relatively homogeneous acoustic medium, increasing only the absolute distance between the skin surface and the stone. In contrast, visceral fat directly surrounds the retroperitoneal space and ureter, creating a highly irregular and dense acoustic interface adjacent to the target stone. Therefore, the substantial nonlinear scattering and local energy dissipation that may compromise ESWL efficacy are likely to arise primarily from the complex periureteral environment represented by visceral fat, rather than from superficial adipose tissue (subcutaneous fat). Overall, these findings suggest that ESWL outcomes are determined not only by intrinsic stone properties but also by the spatial distribution and acoustic characteristics of surrounding body composition.

This study has several limitations. First, it was a single-center retrospective analysis. Most notably, the temporal testing cohort was constrained by a relatively small sample size (*n* = 57) and a low number of residual stone events (*n* = 8). Although the updated combined model achieved a high apparent AUC of 0.898 in this testing set, the resultant confidence intervals remain wide (95% CI: 0.807, 0.989), reflecting considerable statistical uncertainty regarding the true model performance. The extremely high sensitivity observed in the testing cohort is also a mathematical artifact of the low event count rather than a guarantee of flawless clinical detection. Therefore, prospective, multicenter studies with larger independent cohorts and higher event rates are required to precisely validate our results. Second, Our study is fundamentally based on retrospective imaging data and lacks direct acoustic or biomechanical measurements of shockwave propagation. Actual stone composition data were unavailable for our cohort. While it is biologically plausible that a thick layer of visceral fat might physically attenuate acoustic energy transmission before it reaches a highly heterogeneous (high Radscore) stone, this proposed mechanistic explanation remains purely speculative. The observed statistical interactions may also be confounded by other unmeasured anatomical factors. Future translational studies incorporating direct physical measurements of shockwave attenuation and biomechanical modeling are imperative to validate whether these statistical associations reflect true biophysical causal mechanisms. Third, due to substantial missing data on biochemical parameters and BMI, we were unable to comprehensively evaluate the role of BMI and biochemical biomarkers in predicting residual stones after ESWL. Given the known associations between BMI and body composition, future studies should further investigate the interrelationships among BMI, biochemical markers, and body composition features. Finally, the exact timing of stone clearance after ESWL could not be determined in this study. All patients underwent a standardized imaging evaluation within two weeks post-treatment; however, the absence of precise time-to-event data precluded survival-based analyses and limited our ability to characterize the dynamic process of stone clearance.

## Data Availability

The raw data supporting the conclusions of this article will be made available by the authors, without undue reservation.
