# Supplementary material for: Prediction of residual stones after ESWL for ureteral calculi using deep learning-radiomics-based stone heterogeneity and body composition: an interaction analysis
Source: Front Med (Lausanne). 2026 Oct 14;13:1932507. doi: 10.3389/fmed.2026.1932507 (PMC13713844; doi:10.3389/fmed.2026.1932507)
Supplement: Supplementary file 1 [file Supplementaryfile1.docx]

Supplementary Material

# 1 Supplementary information

**S1.** **Imaging Biomarker Standardization Initiative (IBSI) reporting structure of the study**

| **Patients** | |
| --- | --- |
| Region of interest | Segmentation of the regions of interest (ROIs) was performed using 3D Slicer software (version 5.10.0; available at [www.slicer.org](http://www.slicer.org)). The location of the calculi was identified on axial non-contrast abdominal CT images. Visceral fat, skeletal muscle, and subcutaneous fat were obtained from computed tomography (CT) images spanning T12 to S4. |
| Patient Preparation | No special patient preparation (e.g., fasting) was required. All patients underwent a standard whole-abdomen CT examination in accordance with institutional protocols. |
| Computed tomography (CT) developing agent | No intravenous or oral contrast agents were administered |
| **Acquisition and Reconstruction** | |
| Protocol | CT examination: Contrast-enhanced abdominopelvic CT was performed using a 64-slice CT scanner (LightSpeed VCT, GE). The scan range extended from 2 cm above the diaphragm to the superior margin of the pubic symphysis. Scanning parameters were as follows: tube voltage 120 kV, automatic tube current modulation, pitch 0.8, collimation 128 × 0.625 mm. Thin-slice images were reconstructed from the original data with a slice thickness of 1.25 mm and an interval of 1.25 mm. |
| Scanner type | Image data were acquired using a 64-slice computed tomography scanner (LightSpeed VCT, GE). |
| **Delineation** | |
| Software | 3D Slicer (version 5.10.0; www.slicer.org) |
| ROI definition | In 3D Slicer, regions of interest (ROIs) were defined using manual segmentation. The “Paint” and “Erase” tools were applied slice-by-slice for fine adjustments to ensure accuracy. Body composition was automatically segmented into subcutaneous fat, skeletal muscle, and visceral fat using TotalSegmentator. |
| Number of experts | ROI delineation was performed by a radiologist with 5 years of experience. To assess segmentation consistency, 60 patients were randomly selected from the training set and independently re-segmented by a second radiologist with 15 years of experience |
| Reference image | CT |
| **Radiomics feature extraction** | |
| Software | Python (version 3.9) |
| Package | PyRadiomics (https://github.com/AIM-Harvard/pyradiomics); PyTorch (https://pytorch.org/get-started/locally) |
| Method | Images were resampled to 1 mm³ isotropic voxels using B-spline interpolation, with a bin width of 25. ROI masks were resampled using nearest-neighbor interpolation. Because Hounsfield units (HU) on CT images reflect the absolute physical density of tissue attenuation, global gray-level normalization was disabled to preserve the original physical meaning and absolute zero point of the images. And application of multiple image filters including Wavelet, Laplacian of Gaussian (LoG). All feature classes (First Order, Shape, GLCM, GLRLM, GLSZM, NGTDM, GLDM) were computed on the original and filtered images. |
| Discretization | Uniform (equal-width binning) |
| Bin width | 25 for CT |
| Kernels of the filter | Wavelet: coif1 wavelet basis; LoG: sigma values of 1.0, 2.0, 3.0, 4.0, and 5.0 mm; Square, Square Root, Logarithm, Exponential: pixel-wise intensity transformations (no spatial kernel) |
| Biomarker set | First Order Statistics, Shape (3D), Gray Level Co-occurrence Matrix (GLCM), Gray Level Run Length Matrix (GLRLM), Gray Level Size Zone Matrix (GLSZM), Neighboring Gray Tone Difference Matrix (NGTDM), Gray Level Dependence Matrix (GLDM) |
| Exclusion criteria | ICC smaller than 0.75 |

S2. TRIPOD+AI (Transparent Reporting of a multivariable prediction model for Individual Prognosis Or Diagnosis + Artificial Intelligence

**S3. Nested cross-validation (Nested CV) architecture**

**1.** The training cohort (n = 140) was partitioned using a 10-fold Stratified Cross-Validation.

**2.** Within each of the 10 outer training folds, an independent 3-fold inner cross-validation was executed. This inner loop tuned the hyperparameters for both LASSO regression (optimizing the L1 penalty factor λ over 20 density steps) and SVM-RFE (optimizing the exact number of selected features).

**3.** For each outer fold, the intersection of features selected by the inner LASSO and SVM-RFE models was used to fit a logistic regression classifier. This classifier was evaluated on the unseen outer validation fold, yielding an unbiased mean internal AUC.

**4.** To construct the final predictive model, a global feature selection process (10-fold CV for LASSO, 5-fold CV for SVM-RFE) was executed across the entire training set to lock the optimal features and their weighting coefficients.

**Supplementary Tables**

**Table S1.** **Baseline characteristics of stone-free status and residual stones**

| **Variables** | **Total**  **(n = 257)** | **Stone-Free Status**  **(n = 215)** | **Residual Stone**  **(n = 42)** | **t/χ²/-** | ***P*** |
| --- | --- | --- | --- | --- | --- |
|  |  |  |  |  |  |
| **Age (years)** | 48.54 ± 15.06 | 47.68 ± 14.88 | 52.98 ± 15.41 | -2.10 | 0.037 |
| **Gender (%)** |  |  |  | 7.95 | 0.005 |
| Female | 51 (19.84) | 36 (16.74) | 15 (35.71) |  |  |
| Male | 206 (80.16) | 179 (83.26) | 27 (64.29) |  |  |
| **Hypertension (%)** |  |  |  | 16.59 | <0.001 |
| No | 249 (96.89) | 213 (99.07) | 36 (85.71) |  |  |
| Yes | 8 (3.11) | 2 (0.93) | 6 (14.29) |  |  |
| **Diabetes (%)** |  |  |  | 0.02 | 0.875 |
| No | 243 (94.55) | 204 (94.88) | 39 (92.86) |  |  |
| Yes | 14 (5.45) | 11 (5.12) | 3 (7.14) |  |  |
| **Cardiovascular Disease (%)** |  |  |  | - | 0.126 |
| No | 253 (98.44) | 213 (99.07) | 40 (95.24) |  |  |
| Yes | 4 (1.56) | 2 (0.93) | 2 (4.76) |  |  |
| **Perinephritis (%)** |  |  |  | 1.31 | 0.253 |
| No | 143 (55.64) | 123 (57.21) | 20 (47.62) |  |  |
| Yes | 114 (44.36) | 92 (42.79) | 22 (52.38) |  |  |
| **Hydronephrosis (%)** |  |  |  | 1.24 | 0.265 |
| No | 20 (7.78) | 19 (8.84) | 1 (2.38) |  |  |
| Yes | 237 (92.22) | 196 (91.16) | 41 (97.62) |  |  |
| **Maximum stone diameter (mm)** | 4.96 ± 2.68 | 4.54 ± 1.64 | 7.10 ± 5.00 | -3.29 | 0.002 |
| **Mean stone density (HU)** | 650.91 ± 339.71 | 615.55 ± 292.15 | 831.90 ± 484.73 | -2.80 | 0.007 |
| **Skin-to-stone distance (mm)** | 107.15 ± 15.00 | 107.93 ± 15.17 | 103.14 ± 13.55 | 1.90 | 0.058 |
| **Side of the stone (%)** |  |  |  | 2.23 | 0.135 |
| Left | 132 (51.36) | 106 (49.30) | 26 (61.90) |  |  |
| Right | 125 (48.64) | 109 (50.70) | 16 (38.10) |  |  |
| **Stone location (%)** |  |  |  | 3.13 | 0.209 |
| Lower ureter | 97 (37.74) | 86 (40.00) | 11 (26.19) |  |  |
| Mid ureter | 36 (14.01) | 28 (13.02) | 8 (19.05) |  |  |
| Upper ureter | 124 (48.25) | 101 (46.98) | 23 (54.76) |  |  |
| **Proximal ureter diameter (mm)** | 7.00 ± 2.81 | 6.79 ± 2.39 | 8.05 ± 4.26 | -1.86 | 0.069 |
| **Distal ureteral diameter (mm)** | 5.71 ± 2.22 | 5.37 ± 1.77 | 7.42 ± 3.31 | -3.89 | <0.001 |
| **Ureteral wall thickness**  **(mm)** | 3.85 ± 1.51 | 3.68 ± 1.42 | 4.69 ± 1.67 | -4.07 | <0.001 |
| **Skeletal muscle (cm^3^)** | 3256.34 ± 840.64 | 3317.29 ± 846.41 | 2944.30 ± 744.25 | 2.66 | 0.008 |
| **Visceral fat (cm^3^)** | 3344.71 ± 1380.06 | 3261.18 ± 1395.08 | 3772.32 ± 1228.61 | -2.21 | 0.028 |
| **Subcutaneous fat (cm^3^)** | 4015.78 ± 1913.67 | 3950.79 ± 1976.75 | 4348.50 ± 1528.04 | -1.23 | 0.219 |
| **Radscore** | -1.54 ± 1.84 | -1.94 ± 0.76 | 0.52 ± 3.58 | -4.44 | <0.001 |

**Table S2. Multivariable logistic regression analysis of Nomogram model.**

| **Variable** | ***B*** | ***S.E*** | ***P*** | ***OR*** | ***OR* 95%*CI*** | |
| --- | --- | --- | --- | --- | --- | --- |
|  |  |  |  |  | **Lower** | **Upper** |
| **Ureteral wall thickness** | 0.487 | 0.198 | 0.014 | 1.627 | 1.122 | 2.460 |
| **Skeletal muscle** | -0.002 | 0.001 | 0.002 | 0.998 | 0.997 | 0.999 |
| **Visceral fat** | 0.001 | <0.001 | 0.003 | 1.001 | 1.000 | 1.001 |
| **Radscore** | 1.024 | 0.284 | <0.001 | 2.783 | 1.742 | 5.367 |

**Table S3.** Reclassification and Discrimination Improvement of the Nomogram model Compared with the Clinical-body composition model

| **Metrics** | **Estimate** | **SE** | **Z** | **95% CI** | ***P*** |
| --- | --- | --- | --- | --- | --- |
| **Training** |  |  |  |  |  |
| **Continuous NRI** |  |  |  |  |  |
| Overall NRI | 0.339 | 0.209 | 1.621 | -0.071, 0.750 | 0.105 |
| NRI for events | 0.071 | 0.188 | 0.379 | -0.298, 0.441 | 0.705 |
| Increase for events | 53.6% | - | - | - | - |
| Decrease for events | 36.6% | - | - | - | - |
| NRI for non-events | 0.268 | 0.091 | 2.942 | 0.089, 0.446 | 0.003 |
| Decrease for non-events | 46.4% | - | - | - | - |
| Increase for non-events | 63.4% | - | - | - | - |
| **IDI** |  |  |  |  |  |
| Overall IDI | 0.193 | 0.065 | 2.951 | 0.065, 0.320 | 0.003 |
| Mean change in events | 0.154 | - | - | - | - |
| Mean change in non-events | 0.039 | - | - | - | - |
| **Validation** |  |  |  |  |  |
| **Continuous NRI** |  |  |  |  |  |
| Overall NRI | 0.148 | 0.407 | 0.364 | -0.650, 0.947 | 0.716 |
| NRI for events | 0.333 | 0.385 | 0.866 | -0.421, 1.088 | 0.386 |
| Increase for events | 66.7% | - | - | - | - |
| Decrease for events | 59.3% | - | - | - | - |
| NRI for non-events | -0.185 | 0.134 | -1.385 | -0.447, 0.077 | 0.166 |
| Decrease for non-events | 46.4% | - | - | - | - |
| Increase for non-events | 63.4% | - | - | - | - |
| **IDI** |  |  |  |  |  |
| Overall IDI | 0.225 | 0.165 | 1.363 | -0.099, 0.549 | 0.173 |
| Mean change in events | 0.203 | - | - | - | - |
| Mean change in non-events | 0.023 | - | - | - | - |
| **Testing** |  |  |  |  |  |
| **Continuous NRI** |  |  |  |  |  |
| Overall NRI | -0.107 | 0.370 | -0.289 | -0.833, 0.619 | 0.772 |
| NRI for events | -0.250 | 0.342 | -0.730 | -0.921, 0.421 | 0.465 |
| Increase for events | 37.5% | - | - | - | - |
| Decrease for events | 42.9% | - | - | - | - |
| NRI for non-events | 0.143 | 0.141 | 1.010 | -0.134, 0.420 | 0.312 |
| Decrease for non-events | 62.5% | - | - | - | - |
| Increase for non-events | 57.1% | - | - | - | - |
| **IDI** |  |  |  |  |  |
| Overall IDI | 0.119 | 0.116 | 1.020 | -0.109, 0.346 | 0.308 |
| Mean change in events | 0.102 | - | - | - | - |
| Mean change in non-events | 0.017 | - | - | - | - |

NRI, net reclassification index; IDI, integrated discrimination improvement.

**Supplementary figures**

**Figure S1. Flowchart of case inclusion and exclusion.**

**Figure S2. Top 20 feature stability rankings from 10-fold nested cross-validation (A); unbiased AUC distribution from 10-fold nested cross-validation (B)**

**Figure S3. A) Characterizing the Variations of LASSO Regression Coefficients. B) LASSO regression selects the optimal parameter lambda through cross-validation. The dashed line on the right represents lambda values with average error within ±1 standard deviation, indicating improved model performance.**
